# Fulminant myocarditis: COVID or not COVID? Reinfection or co-infection?

**DOI:** 10.2217/fca-2020-0237

**Published:** 2021-02-22

**Authors:** Ramya Yeleti, Maya Guglin, Kashif Saleem, Sasikanth V Adigopula, Anjan Sinha, Smrity Upadhyay, Jeffrey E Everett, Kareem Ballut, Sarada Uppuluri, Roopa A Rao

**Affiliations:** ^1^College of Osteopathic Medicine, Marion University, 3200 Cold Spring Rd, Indianapolis, IN 46222, USA; ^2^Patient Author, Carmel, Indiana, IN 46032, USA; ^3^Division of Cardiology, Krannert Institute of Cardiology at Indiana University School of Medicine, 1800 N Capital Ave, Indianapolis, IN 46202, USA; ^4^Division of Cardiothoracic Surgery, Indiana University School of Medicine, 1701 N Senate Blvd, Indianapolis, IN 46202, USA; ^5^Division of Cardiology, Community Heart & Vascular Hospital, 8075 Shadeland Ave, Indianapolis, IN 46250, USA; ^6^Division of Infectious Disease, Indiana University School of Medicine, 545 Barnhill Dr. Emerson Hall 305, Indianapolis, IN 46202, USA

**Keywords:** cardiogenic shock, coronavirus disease-19, fulminant myocarditis, SARS-CoV-2, VA ECMO

## Abstract

We describe a unique case of fulminant myocarditis in a patient with presumed SARS-CoV-2 reinfection. Patient had initial infection 4 months backand had COVID-19 antibody at the time of presentation. Endomyocardial biopsy showed lymphocytic myocarditis, that is usually seen in viral myocarditis. The molecular diagnostic testing of the endomyocardial biopsy for cardiotropic viruses was positive for Parvovirus and negative for SARS-CoV-2. Authors highly suspect co-infection of SARS-CoV-2 and Parvovirus, that possibly triggered the immune cascade resulting in fulminant myocarditis. Patient was hemodynamically unstable with ventricular tachycardia and was supported on VA ECMO and Impella CP. There was impressive recovery of left ventricular function within 48 hours, leading to decannulation of VA ECMO in 72 h. This unique case was written by the survivor herself.

## Case presentation

**Figure 1.**
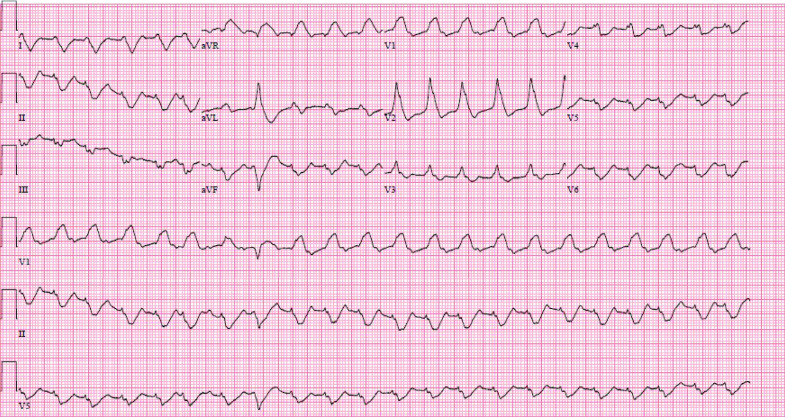
Electrocardiogram showing ventricular tachycardia upon presentation.

We describe a case of a 25-year-old medical student with no significant past medical history except for vitiligo. She had coronavirus disease-19 (COVID-19)-like symptoms 4 months back after close contact with COVID-19. SARS-CoV-2 IgG antibody was positive 3 months prior to presentation. She presented to the emergency room this time with fever, abdominal pain, fatigue and vomiting for 2 days. Upon presentation she was found to be in ventricular tachycardia (Figure 1). Echocardiogram showed biventricular failure and left ventricular ejection fraction (LVEF) of 5–10%. She was defibrillated externally to normal sinus rhythm, however, continued to be hemodynamically unstable. Bilateral Impellas (Impella CP and Impella RP (Abiomed, MA, USA) were placed emergently. Despite this patient continued to deteriorate hemodynamically. So emergent peripheral veno arterial extracorporeal membrane oxygenator (VA-ECMO) was placed with removal of RP Impella. Impella CP was retained for left ventricular venting.

**Figure 2.**
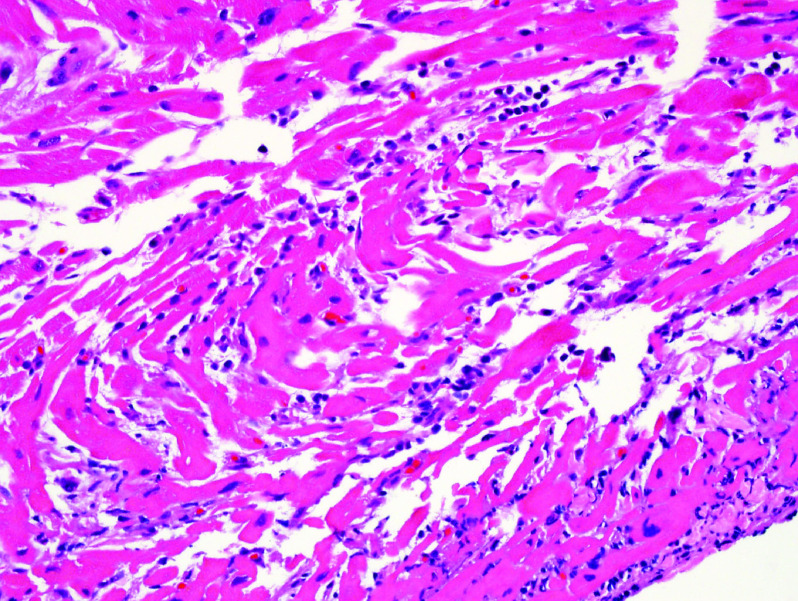
Endomyocardial biopsy showing lymphocytic myocarditis.

SARS-CoV-2 PCR was persistently positive on days 1, 7 and 10 of hospitalization. SARS-CoV-2 IgG antibody was checked due to history of COVID infection, which came back positive on day 1. Inflammatory markers and troponin were elevated (Table 1). For further work up of fulminant myocarditis (FM) endomyocardial biopsy was done. Biopsy showed lymphocytic infiltrates consistent with viral myocarditis (Figure 2). Small foci of myocardial necrosis were seen as well. Immunohistochemistry was negative. The molecular diagnostic testing of the endomyocardial biopsy for cardiotropic viruses was positive for Parvovirus-19. SARS-CoV 2 PCR was negative. The cycle threshold (ct) value for Parvovirus was 27 ct (value less than 29 ct indicating high viral load). The electron microscopy of the biopsy did not show any viral inclusion particles. Serum Parvovirus antibody testing IgM was negative (0.42 Index value) and IgG was positive (7.7 Index Value). Rest of the viral panel was negative. Autoimmune panel was positive for anti-Ro antibody (5.1 test value). Chest X-ray was clear indicating no pulmonary involvement.

**Table 1.** Showing laboratory parameters.

Laboratory test	Patient value	Normal reference range
Troponin	10.65 ng/ml	<0.03 ng/ml
BNP	117 pg/ml	0–100 pg/ml
IL-6	16.5 pg/ml	0–6.3 pg/ml
Ferritin	935.1 ng/ml	10–106 ng/ml
CRP	3.0 mg/dl	<1.0 mg/dl
Creatinine	1.05 mg/dl	0.60–1.20 mg/dl
ALT	34 Units/l	7–52 Units/l

## Management

After initial stabilization on mechanical support, intravenous methylprednisolone 1 gm was started on day 1. On day 2, moderate pericardial effusion (Supplementary Video 1) was noted, and pericardial window was done. At this time COVID test results came back positive along with positive antibody. Due to the severity of the hemodynamic compromise, she was treated with Remdesivir and convalescent plasma. Echocardiogram done the very next day (48 hours of presentation) showed impressive recovery of biventricular function with near normal LVEF (Supplementary Video 2). After another 24 hours of hemodynamic stability, patient was successfully decannulated from VA ECMO and was maintained on Impella support alone.

The next day transesophageal echocardiogram was done prior to the removal of the Impella. Severe mitral regurgitation was noted due to the flail and torn A3 component of anterior mitral valve. Mitral valve repair was immediately performed with 6 gortex neo-chords to A3 of anterior mitral valve and annuloplasty ring. Separation from cardiopulmonary bypass was delayed due to biventricular failure, however there was impressive recovery of LVEF to normal once again in the operating room. Patient was extubated the next day.

**Figure 3.**
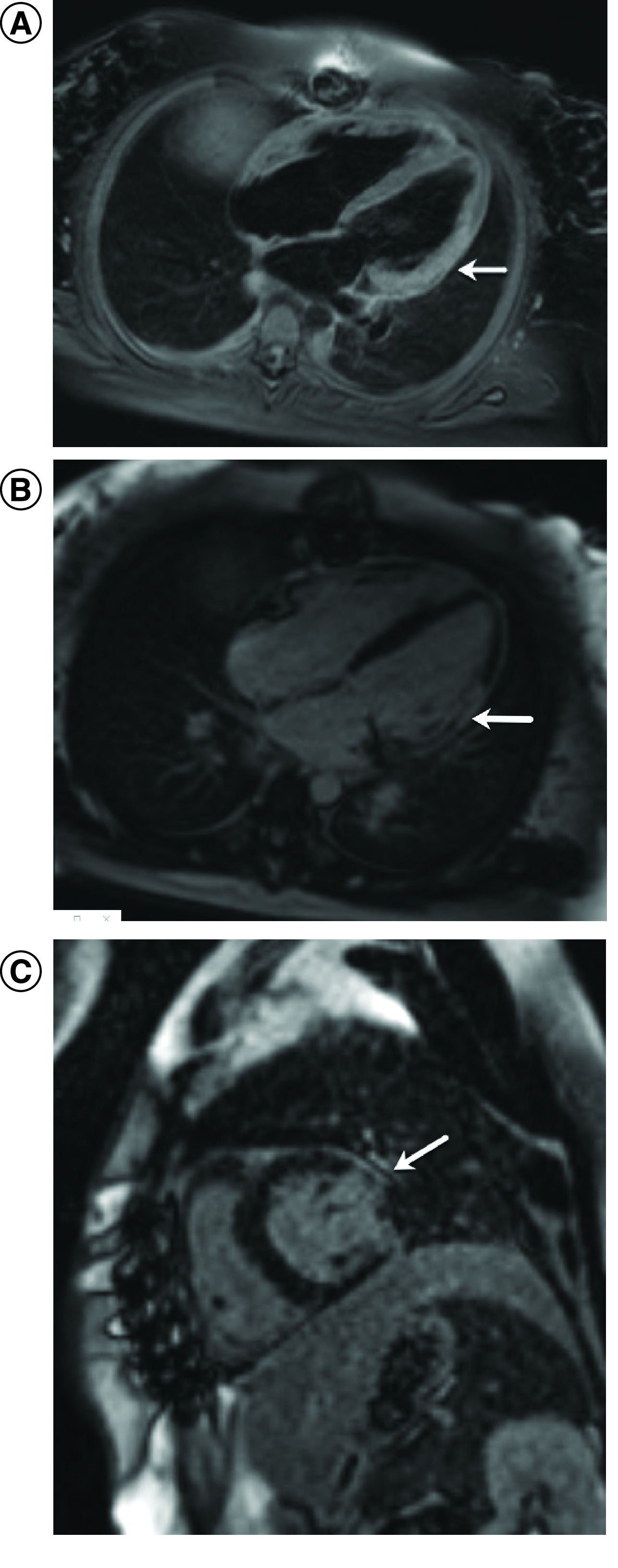
Cardiac MRI findings. **(A)** CMR showing abnormal increased T2 signal (80 ms) in the lateral wall of the left ventricle indicating myocardial edema. **(B)** Transmural delayed gadolinium enhancement of basal and mid anterolateral, inferolateral segments. **(C)** Short-axis view of the CMR showing transmural delayed gadolinium enhancement.

Cardiac MRI (CMR) done prior to discharge showed transmural late gadolinium enhancement of basal-mid anterolateral and inferolateral segments (Figure 3). Patient was continued on high dose steroids and was discharged to home in stable condition and normal LVEF (Supplementary Video 3) on day 13.

At the 4-month follow-up, the patient continues to do well on medical therapy with preservation of LVEF. Currently, she is on a slow steroid wean.

## Discussion

This case is unique for various reasons. SARS-CoV-2 PCR was positive after 4 months of initial infection, raising the possibility of reinfection. According to CDC patients can get reinfection after 3 months of initial infection. However, the patient also had Positive SARS-CoV-2 IgG antibody at the time of presentation. SARS-CoV-2 IgG antibody is thought be highly protective in preventing reinfection. Ours is the first case to isolate Parvovirus genome in the myocardial tissue in a patient with COVID-19. FM as the initial presentation of SARS-CoV-2 infection, requiring ECMO for hemodynamic support is rare. The other distinctive features include rapid recovery of FM within 48 hours of presentation and ECMO decannulation in 72 hours. This case report is quite unique as one the authors of the case report is the survivor herself.

Cardiovascular involvement from SARS-CoV-2 although is widely reported, in general is uncommon. FM in COVID patients has been limited to case reports [1]. The exact mechanism of myocarditis in COVID patients is not clear. SARS-CoV-2 is not thought to be a cardiotropic virus [2]. Isolation of the virus from the cardiomyocyte in the autopsy or biopsy specimen has not been possible [2]. However viral particles have been found in cardiomyocyte in one pediatric patient [3] and in the myocardial macrophage in another case [4]. Alternatively, virus can trigger inflammatory response in the form of SIRS, lymphopenia, macrophage activation and cytokine storm releasing IL-6, IL-10 and TNF-α which can cause myocarditis [5].

Co-infection with another virus or bacteria is a known common phenomenon resulting in myocarditis. Ours is the first case to demonstrate parvovirus in the endomyocardial biopsy in a patient otherwise with presumed COVID myocarditis. Parvovirus is a cardiotropic virus and FM has been described [6]. Shah *et al.* noticed high coinfection rate (22.4%) in COVID-19 patients [7]. Probably we can postulate that infection with SARS-CoV-2 and Parvovirus co-infection triggered immune cascade and lead to FM. In this patient IgM for parvo virus was negative and IgG was positive indicating past infection. IgG antibody production usually occurs 18–24 days after the infection and can persist for years.

It is not very clear if the patient contracted reinfection as she was persistently positive for PCR in the presence of SARS-CoV-2 antibody. However, it is currently not known if or how antibody testing results correlate with immunity to SARS-CoV-2 infection. COVID reinfection is being reported as early as 4 weeks from initial infection and the second infection can be more severe [8,9]. Alternatively, patients can persistently test positive despite clearing the infection, partly due to presence of viral particles.

There is a prevalent theory of a link between acute myocarditis (AM) and autoimmune disease; the association is seen in about 7.2% of AM patients [10]. The patient was positive for anti-Ro antibodies, had significant inflammation on CMR and had history of vitiligo which can be associated other autoimmune conditions. These factors were taken into consideration in initiating glucocorticoid therapy. Since her LVEF recovered rapidly, concomitant therapy with azathioprine, cyclosporine and Mycophenolate mofetil were not considered due to lack of convincing evidence from a risk versus benefit analysis. Notably, the patient received convalescent plasma immunoglobulin during her stay. Research regarding Immunoglobin as AM treatment is well described in children. The Immunoglobulin therapy may have a role in AM as cardiac autoantibodies are frequently seen in myocarditis. One may consider using Immunoglobulin therapy especially in hemodynamically unstable patient as a treatment modality [11].

## Conclusion

FM with cardiogenic shock can be expected in COVID patients and requires a full extent of hemodynamic support along with anti-viral treatment. This case raises the impact of possible co-infection in COVID patients. This case also highlights the diagnostic challenges that we face in the era of pandemic.

**Summary points**

- Cardiovascular complications although are uncommon in SARS-CoV-2 infection, is increasingly being reported.
- Fulminant Myocarditis can be seen in patients with SARS-CoV-2 infection.
- Full hemodynamic support when appropriate should be considered, as there is chance of recovery of left ventricular function.
- Co-infection should be in the differential when treating patients with SARS-CoV-2 infection.
